# Effectiveness and safety of capecitabine, irinotecan and panitumumab in advanced colorectal cancer

**DOI:** 10.3389/fonc.2023.1138357

**Published:** 2023-04-06

**Authors:** Pui Lam Yip, Wai Him Brian Fung, Francis Ann Shing Lee, Chak Fei Lee, Natalie Sean Man Wong, Shing Fung Lee

**Affiliations:** ^1^ Department of Clinical Oncology, Tuen Mun Hospital, New Territories West Cluster, Hospital Authority, Hong Kong, Hong Kong SAR, China; ^2^ Department of Radiation Oncology, National University Cancer Institute, National University Hospital, Singapore, Singapore; ^3^ Department of Radiology and Nuclear Medicine, Tuen Mun Hospital, New Territories West Cluster, Hospital Authority, Hong Kong, Hong Kong SAR, China; ^4^ Department of Pharmacy, Tuen Mun Hospital, New Territories West Cluster, Hospital Authority, Hong Kong, Hong Kong SAR, China

**Keywords:** colorectal neoplasms, drug therapy, anti-EGFR, survival, toxicity

## Abstract

**Introduction:**

Capecitabine, irinotecan, and panitumumab (CAPIRI-P) is a controversial regimen for metastatic colorectal cancer, with concerns regarding the efficacy and toxicity. However, its toxicity profile has been improved with dose reduction, and concerns regarding efficacy have been extrapolated from other trials. This retrospective study reports the real-world effectiveness and safety of modified CAPIRI-P (mCAPIRI-P).

**Material and methods:**

Advanced colorectal cancer patients receiving mCAPIPI-P in the first-line setting between July 2019 and December 2021 were analyzed. The progression-free survival on treatment (PFS_OT_) and overall survival (OS) were estimated using the Kaplan–Meier method, and the association with clinical and disease factors was analyzed using the Cox regression model. Serial changes in carcinoembryonic antigen (CEA) level and treatment toxicity were also evaluated.

**Results:**

A total of 106 patients were included, of whom 97 (92%) and 31 (29%) had left-sided primary and unresectable liver-only disease, respectively. The median PFS_OT_ and OS were 15.4 (95% CI 12.5–18.3) and 25.5 (95% CI 17.6–33.4) months, respectively. Sixteen (51.6%) and 10 (32.3%) liver-only disease patients underwent secondary liver treatment and R0 resection, respectively. In multivariable Cox regression, CEA responders (PFS_OT_: HR 0.53) and CEA normalization (PFS_OT_: HR 0.27; OS: HR 0.28) were independent favorable prognostic factors for PFS_OT_ and OS. Grade ≥3 toxicity rate was 43%, mainly related to uncomplicated hematological toxicities.

**Conclusion:**

The real-world data show that mCAPIRI-P is safe and effective as the first-line treatment regimen for RAS wild-type advanced colorectal cancer and warrants further study.

## 1 Introduction

Combination chemotherapy with fluoropyrimidine and oxaliplatin or irinotecan is the standard backbone in the first-line treatment for metastatic colorectal cancer (1, 2). The addition of anti-epidermal growth factor receptor (EGFR) antibodies to doublet chemotherapy has improved the treatment outcome (3–5). Although many landmark trials have studied the combination of panitumumab with infusional 5-fluorouracil (5-FU) and oxaliplatin (5–8), its effectiveness when added to irinotecan and capecitabine (CAPIRI) has not been reported. Moreover, CAPIRI with an anti-EGFR antibody is a much debated regimen (1, 9), with concerns regarding its efficacy and toxicity. In our institute, a modified three-week capecitabine, irinotecan, and panitumumab (mCAPIRI-P) has been widely adopted due to the COVID-19 pandemic.

This retrospective cohort study reports the effectiveness and safety of mCAPIRI-P regimen on consecutive RAS wild-type (WT) advanced colorectal cancer patients and carcinoembryonic antigen (CEA) kinetics.

## 2 Materials and methods

Patients receiving combination chemotherapy with capecitabine, irinotecan, and panitumumab (mCAPIRI-P) from July 2019 to December 2021 at Tuen Mun Hospital were evaluated. Patients were included if chemotherapy was administered for RAS WT unresectable locally advanced or metastatic colorectal cancer in the first-line setting. The patient was excluded if panitumumab was started later than the fifth cycle of chemotherapy and/or if the patient had no active disease, for example, upfront metastectomy was performed.

Patient demographics, chemotherapy dose and record, treatment outcomes and toxicity, and CEA levels were retrieved from electronic medical records and hospital records. In our institute, the mCAPIRI-P regimen comprised three-week cycles of oral 800 mg/m^2^ capecitabine twice daily on days 1–14, intravenous 200 mg/m^2^ irinotecan on day 1, and intravenous 9 mg/kg panitumumab on day 1. Patients were monitored for clinical symptoms, tumor markers, and radiological findings. For patients with liver-only disease, the management of liver metastasis was reviewed with hepatobiliary surgeons in regular multidisciplinary team meetings. Chemotherapy was continued until disease progression or in some patients, drug holidays. A drug holiday with subsequent resumption was offered at the physician’s discretion and patient preference.

### 2.1 Effectiveness

Effectiveness of the chemotherapy was assessed by progression-free survival on treatment (PFS_OT_) and overall survival (OS). For survival endpoints, patients were censored at the last follow-up visit. PFS_OT_ is the time from the date of the first cycle of chemotherapy to the date of documented disease progression during active treatment or death. Disease progression during drug holidays was not considered true progression; thus, it was not a PFS_OT_ event. The patient would resume mCAPIRI-P until progression on treatment or another drug holiday. OS is the time interval from the date of the first cycle of chemotherapy until death from any cause. A subgroup of patients receiving conversion chemotherapy for liver-only disease were assessed for the treatment response in the liver by Response Evaluation Criteria in Solid Tumors (RECIST) 1.1 (10), secondary liver treatment, and R0 resection (defined as no microscopic or macroscopic residual tumor). Disease progression was determined by the treating physician with supporting evidence from clinical symptoms, radiological findings, and/or biomarkers.

### 2.2 CEA kinetics

CEA ≤ 5 ng/mL was considered normal. CEA response rate (11) is the percentage reduction of CEA from the initial value to the nadir after chemotherapy. CEA responders are patients having ≥75% CEA response rate (11).

### 2.3 Toxicities

Toxicities of chemotherapy were graded according to the National Cancer Institute Common Terminology Criteria for Adverse Events (CTCAE) v4 (12).

### 2.4 Statistical analysis

Descriptive statistics for demographics, follow-up duration, and characteristic prevalence were generated. Continuous variables were presented as medians with interquartile ranges (or means and standard deviations).

We estimated PFS_OT_ and OS using the Kaplan–Meier method (13, 14). Univariable Cox regression was performed to evaluate the associations between variables (demographic factors, clinical characteristics, and CEA kinetics) and PFS_OT_ and OS. Variables with a significant association (p<0.05) were selected and tested using multivariable Cox regression (15). Variables linked to CEA kinetics (namely baseline normal CEA, CEA normalization and CEA responder) were added individually to the Cox models. A two-sided p<0.05 was used to determine statistical significance. All statistical analyses were performed using IBM SPSS Statistics for Windows version 21 (IBM Corp.; Armonk, N.Y., USA).

The study protocol was approved by the Research Ethics Committee of the New Territories West Cluster, Hong Kong Hospital Authority (reference no. NTWC/REC/21037).

## 3 Results

This study analyzed data from 106 consecutive patients. The mean age was 63 years, and the median follow-up period was 16 months (IQR 11–23 months). Among them, 97 (91.5%) had left-sided colorectal cancer, 97 (91.5%) were treated with palliative intent for metastatic disease, and 31 (29.3%) had unresectable liver-only disease. During analysis, 91.5% of patients completed at least one treatment course. 26 (24.5%) decided for drug holiday after the first treatment course. A median of nine cycles of chemotherapy was administered (**Table 1**).

**Table 1:** Baseline characteristics of the study cohort, Hong Kong, 2019–2021 (N = 106).

Characteristics	All Patients (N=106)
Age	
Mean (SD, range)	63 (9, 23-79)
≤70, n (%)	88 (83.0)
>70, n (%)	18 (17.0)
Sex	
Male, n (%)	80 (75.5)
Female, n (%)	26 (24.5)
KPS	
≥90, n (%)	62 (58.5)
80, n (%)	36 (34.0)
≤70, n (%)	8 (7.5)
Treatment intent	
Locally Advanced, unresectable, n (%)	5 (4.7)
Upfront resectable metastasis, n (%)	4 (3.8)
Metastatic, palliative, n (%)	97 (91.5)
Primary tumor location	
Right, n (%)	9 (8.5)
Left, n (%)	45 (42.5)
Rectosigmoid junction/rectum, n (%)	52 (49.1)
Liver-only disease	
All	34 (32.1)
Upfront resectable, n (%)	3 (2.8)
Unresectable, n (%)	31 (29.3)
Chemotherapy	
Completed/on drug holiday, n (%)	97 (91.5)
Ongoing, n (%)	9 (8.5)
Total Number of cycles, Median (IQR)	9 (7-14)
Any dose reduction	
Capecitabine	
Omit, n (%)	3 (2.8)
≥70%, n (%)	93 (87.7)
<70%, n (%)	10 (9.4)
Irinotecan	
Omit	2 (1.9)
≥70%, n (%)	98 (92.5)
<70%, n (%)	6 (5.7)
Panitumumab	
Omit	0
≥70%, n (%)	102 (96.2)
<70%, n (%)	4 (3.8)
Any termination toxicity, n (%)	5 (4.7)
Unplanned hospitalization	
Any, n (%)	44 (41.5)
Treatment related, n (%)	15 (14.2)
Disease related, n (%)	19 (17.9)
Others, n (%)	10 (9.4)

KPS, Karnofsky performance status; SD, standard deviation.

### 3.1 Survival

**Figure 1:**
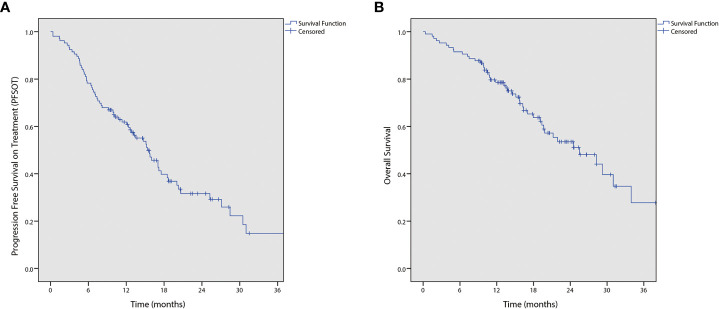
Kaplan-Meier curves of **(A)** progression free survival on treatment (PFSOT) and **(B)** overall survival (OS).

During analysis, 68 (64.2%) patients had PFS_OT_ events. Moreover, three patients documented progressive disease on drug holiday; among them, two decided to continue drug holiday and one died before resuming chemotherapy. The median PFS_OT_ was 15.4 months (95% CI 12.5–18.3). The 1-year PFS_OT_ was 61.9% (95% CI 52.5%–71.3%). Furthermore, 46 (43.4%) deaths occurred, and the median OS was 25.5 months (95% CI 17.6–33.4; **Figure 1**).

### 3.2 Conversion chemotherapy

In total, 31 patients had unresectable liver-only disease (**Table 2**). Two (6.5%), 20 (64.5%), 3 (9.7%), and 4 (12.9%) achieved complete response (CR), partial response (PR), stable disease (SD), and progressive disease (PD) in the liver, respectively. The objective response rate was 71%. The two patients with CR were put on observation and were not offered liver treatment. Sixteen (51.6%) patients underwent secondary liver treatment. The R0 resection rate was 32.3%.

**Table 2:** Patients who received conversion chemotherapy for liver-only disease, 2019–2021 (N = 31).

Characteristics	N=31
Number of liver metastasis	
1-3, n (%)	9 (29.0)
4-10, n (%)	10 (32.3)
11-20, n (%)	7 (22.6)
>20, n (%)	5 (16.1)
Treatment response	
Complete response, n (%)	2 (6.5)
Partial response, n (%)	20 (64.5)
Stable disease, n (%)	3 (9.7)
Progressive disease, n (%)	4 (12.9)
Not available, n (%)	2 (6.5)
Local treatment	
Any, n (%)	16 (51.6)
Hepatectomy only, n (%)	0
Segmentectomy only, n (%)	5 (31.3)
Wedge resection only, n (%)	4 (25.0)
Ablation only, n (%)	1 (6.3)
Combined, n (%)	6 (37.5)

### 3.3 CEA kinetics

In total, 93 (87.7%) patients had baseline elevated CEA. Among them, CEA was normalized in 40 (43.0%) patients. The median time to nadir was 138.50 days (93.50–196.25). The median CEA response rate was 88.9% (67.3–96.9). Moreover, 62 (68.9%) patients were CEA responders with ≥75% CEA response rate.

**Figure 2:**
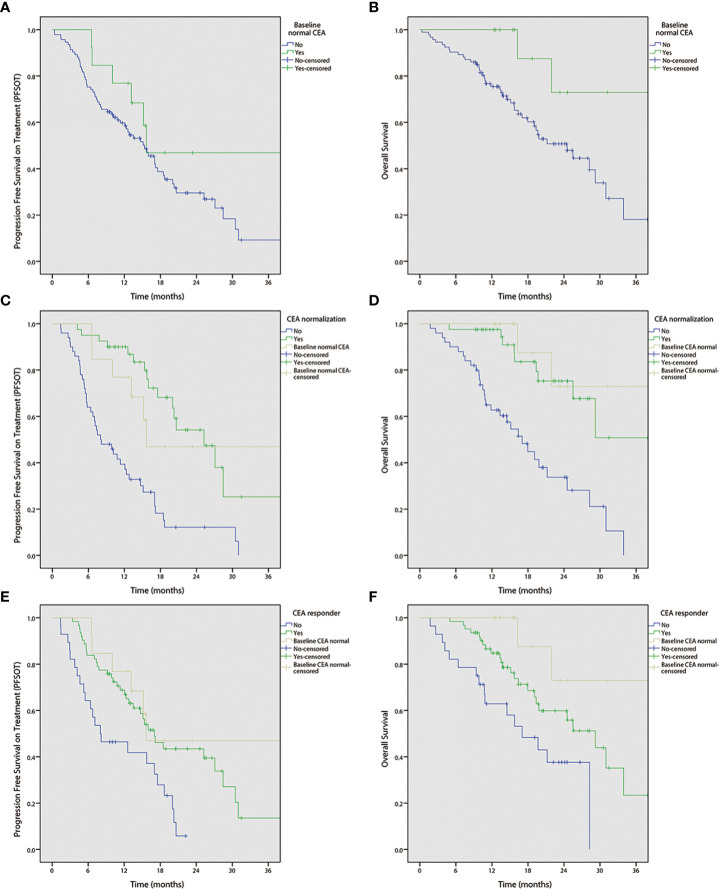
Kaplan-Meier curves of progression free survival on treatment (PFSOT) and overall survival (OS) by carcinoembryonic antigen (CEA) kinetics. **(A)** PFSOT by baseline normal CEA, **(B)** OS by baseline normal CEA. **(C)** PFSOT by CEA normalization. **(D)** OS by CEA normalization. **(E)** PFSOT by CEA responder. **(F)** OS by CEA responder.

Univariable analyses (**Figure 2** and **Tables 3**, **4**) reported that liver-only disease, CEA normalization, and CEA responder were both OS and PFS_OT_ predictors. Additionally, Karnofsky performance status (KPS) and baseline normal CEA levels were OS predictors. However, multivariable analyses (**Tables 3**, **4**) reported that CEA responders (PFS_OT_: HR 0.53, CI 0.31–0.92; OS: HR 0.53, CI 0.27–1.04) and CEA normalization (PFS_OT_: HR 0.27, CI 0.15–0.48; OS: HR 0.28, CI 0.13–0.58) remained independent predictors of better OS and PFS_OT_.

**Table 3:** Univariable and multivariable analyses of prognostic factors for progression-free survival on treatment, Hong Kong, 2019–2021 (N = 106).

Variables	Progression-free survival on treatment					
	Univariable analysis		Multivariable analysis			
			Model 1^a^		Model 2^a^	
	HR (95% CI)	*P*	HR (95% CI)	*P*	HR (95% CI)	*P*
**Age** (continuous per 1 year)	0.98 (0.96–1.01)	0.25	–	–	–	–
**Sex** (female vs. male)	1.22 (0.71–2.10)	0.47	–	–	–	–
**KPS** (>80 vs. ≤80)	0.67 (0.41–1.08)	0.10	–	–	–	–
**Stage (metastatic vs. non-metastatic)**	1.08 (0.34-3.44)	0.90	–	–	–	–
**Treatment Intent** Upfront resectable metastasis vs. locally advanced unresectable Metastatic palliative vs. locally advanced unresectable	0.20 (0.02–2.02) 1.13 (0.35–3.60)	0.17 0.84	–	–	–	–
**Primary tumor location** (left vs. right)	0.64 (0.26–1.62)	0.35	–	–	–	–
**Liver-only disease** (yes vs. no)	0.40 (0.22–0.75)	0.004	0.50 (0.26–0.95)	0.03	0.49 (0.26–0.90)	0.02
**Baseline normal CEA** (yes vs. no)	0.49 (0.21–1.15)	0.49	–	–	–	–
**CEA normalization** Yes vs. no Baseline normal CEA vs. no	0.26 (0.14–0.45) 0.27 (0.11–0.65)	<0.001 0.003	–	–	0.27 (0.15–0.48) 0.29 (0.12–0.71)	<0.001 0.006
**CEA responder** Yes [response rate ≥75%] vs. no [CEA response rate <75%]) Baseline normal CEA vs. no [CEA response rate <75%])	0.45 (0.26–0.77) 0.29 (0.11–0.72)	0.004 0.008	0.53 (0.31–0.92) 0.34 (0.13–0.86)	0.03 0.02	–	–

CEA, carcinoembryonic antigen; CI, confidence interval; HR, hazard ratio; KPS, Karnofsky performance status.

a Factors having P ≤0.05 were selected into the multivariable model. Variables linked to CEA kinetics (i.e. baseline normal CEA, CEA normalization and CEA responder) were added individually to the Cox models.-, The variable is not applicable for the multivariable models.

**Table 4:** Univariable and multivariable analyses of prognostic factors for overall survival, Hong Kong, 2019–2021 (N = 106).

Variables	Overall survival							
	Univariable analysis		Multivariable analysis					
			Model 1^a^		Model 2^a^		Model 3^a^	
	HR (95% CI)	*P*	HR (95% CI)	*P*	HR (95% CI)	*P*	HR (95% CI)	*P*
**Age** (continuous per 1 year)	0.98 (0.95–1.01)	0.21	–	–	–	–	–	–
**Sex** (female vs. male)	1.60 (0.85–3.03)	0.15	–	–	–	–	–	–
**KPS** (>80 vs. ≤80)	0.51 (0.28–0.93)	0.03	0.64 (0.35–1.17)	0.15	0.72 (0.39–1.35)	0.31	0.57 (0.30–1.06)	0.08
**Stage (metastatic vs. non-metastatic)**	2.78 (0.38-20.19)	0.31	–	–	–	–	–	–
**Treatment Intent** Upfront resectable metastasis vs. locally advanced unresectable Metastatic palliative vs. locally advanced unresectable	0.74 (0.04–12.51) 2.90 (0.40–21.13)	0.84 0.29	–	–	–	–	–	–
**Primary tumor location** (left vs. right)	0.76 (0.23–2.47)	0.65	–	–	–	–	–	–
**Liver-only disease** (yes vs. no)	0.35 (0.16–0.77)	0.01	0.40 (0.19–0.86)	0.02	0.48 (0.22–1.04)	0.06	0.50 (0.22–1.10)	0.08
**Baseline normal CEA** (yes vs. no)	0.20 (0.05–0.85)	0.03	0.27 (0.06–1.13)	0.07	–	–	–	–
**CEA normalization** Yes vs. no Baseline normal CEA vs. no	0.25 (0.12–0.51) 0.11 (0.03–0.49)	<0.001 0.004	–	–	0.28 (0.13–0.58) 0.15 (0.03–0.68)	0.001 0.01	–	–
**CEA responder** Yes [response rate ≥75%] vs. no [CEA response rate <75%]) Baseline normal CEA vs. no [CEA response rate <75%])	0.48 (0.25–0.92) 0.12 (0.03–0.55)	0.03 0.01	–	–	–	–	0.53 (0.27–1.04) 0.18 (0.04–0.84)	0.06 0.03

CEA, carcinoembryonic antigen; CI, confidence interval; HR, hazard ratio; KPS, Karnofsky performance status.

a Factors having P ≤0.05 were selected into the multivariable model. Variables linked to CEA kinetics (i.e. baseline normal CEA, CEA normalization and CEA responder) were added individually to the Cox models.-, The variable is not applicable for the multivariable models.

### 3.4 Toxicities

Ten (9.4%), six (5.7%), and four (3.8%) patients required dose reduction to <70% of the intended dose of capecitabine, irinotecan, and panitumumab, respectively. Unplanned hospitalization occurred in 44 patients, of whom 15 (14.2%) and 19 (17.9%) were due to treatment toxicity and disease-related symptoms or complications, respectively. Five (4.7%) patients terminated the treatment due to toxicity (**Table 1**). Grade 3-4 toxicities were observed in 46 (43.4%) patients, of whom 32.1%, 16.0%, and 11.3% experienced uncomplicated neutropenia, uncomplicated leukopenia, and diarrhea, respectively. Febrile neutropenia occurred in 3.8%. No grade 5 toxicity was observed (**Table 5**).

**Table 5:** Grade 3-4 treatment toxicities, Hong Kong, 2019–2021 (N = 106).

Toxicities	N (%)
Leukopenia	17 (16.0)
Neutropenia	34 (32.1)
Thrombocytopenia	3 (2.8)
Febrile neutropenia	4 (3.8)
Rash	2 (1.9)
Paronychia	4 (3.8)
Stomatitis	2 (1.9)
Hand-foot syndrome	4 (3.8)
Diarrhea	12 (11.3)
Nausea/vomiting	6 (5.7)
Hypomagnesemia	6 (5.7)

## 4 Discussion

To the best of our knowledge, this is the first report on the effectiveness and safety of mCAPIRI-P in a three-week schedule. Due to the availability of public funding for panitumumab and the COVID-19 pandemic, mCAPIRI-P was offered to all left-sided RAS WT and selected right-sided RAS WT colorectal cancer patients as a first-line treatment choice. During the study period, only a small proportion of highly selected patients received triplet chemotherapy. Our consecutive data are representative of the mCAPIRI-P treatment outcomes in a real-world setting.

PFS_OT_ was studied because intermittent therapy has been widely adopted in local practice. The median PFS_OT_ (15.4 months) and 1-year PFS_OT_ (61.9%) are similar to those reported in other landmark trials including PARADIGM (7) (12.9 months), PRIME (5) (10 months), and CRYSTAL (3) (9.9 months). It is also comparable to the results of IMPROVE (16) trial, which also studied PFS_OT_ (median PFS_OT_ 17.6 months, 1-year PFS_OT_ 61.3%; **Supplementary Table 1**). The OS data of this study were not mature during the analysis. Furthermore, mCAPIRI-P was also proved to be an efficacious regimen in the conversion setting for liver-only disease. The secondary liver treatment rate (51.6%) and R0 resection rate of this study were similar to those of other major reports, including CELIM (17), Ye et al. (18), TRIPLETE (8), and VOLFI (6) (**Supplementary Table 1**).

mCAPIRI-P use raised no additional safety concerns in terms of toxicity. the rate of grade ≥3 diarrhea was 11.3% which is similar to that found in other trials with 5-FU (19) or oxaliplatin (5) backbones. Uncomplicated grade≥3 leukopenia and neutropenia rates were higher than those reported in other irinotecan-based trials (19, 20). This is probably because the interim blood cell count was frequent, such as at the second week. Nonetheless, the rate of febrile neutropenia (3.8%) remained similar to other landmark trials (5, 20). Notably, the incidence of severe skin/nail toxicity (<4%) was low, which could be attributed to the routine use of empirical oral doxycycline, topical steroid, and emulsifying lotion, as suggested in the STEPP (21) and JSTEPP (22). Pharmacogenetic tests are not routinely performed for proactive dose reduction. Some patients who experienced significant toxicities were later found to have dihydropyrimidine dehydrogenase (DPD) deficiency or UGT1A1 polymorphism.

mCAPIRI-P is not widely used. First, although CAPIRI combination demonstrated tolerable toxicity in European trials (23, 24), it was suggested to be too toxic in other trials (25, 26). Thus, the National Comprehensive Cancer Network (NCCN) guideline does not recommend CAPIRI (1). However, the toxicity profile was much improved after dose reduction (a.k.a. modified CAPIRI (mCAPIRI)), as demonstrated in the phase III AXEPT (20) and other trials (27, 28). Second, the European Society for Medical Oncology (9) and NCCN guidelines (1) do not recommend using anti-EGFR antibodies in combination with capecitabine-based regimens. This recommendation was based on MRC-COIN (4) indicating that cetuximab in combination with capecitabine and oxaliplatin had worse outcomes than 5-FU-based chemotherapy in a subgroup analysis, which, according to the authors, could be related to the increased toxicity with subsequent reduction in drug dose and exposure. Such toxicity was again improved after capecitabine dose reduction. Notably, there is no direct evidence suggesting inferior efficacy or toxicity of capecitabine and anti-EGFR antibodies using an irinotecan backbone. Indeed, the use of CAPIRI and anti-EGFR antibodies has been supported in previous phase II studies (23, 29). Third, panitumumab has been licensed as a biweekly regimen (30) instead of the three-week regimen in this study. The three-week regimen was supported in a phase I study (31), which indicated that three-week 9 mg/kg panitumumab and biweekly 6 mg/kg panitumumab have similar exposure and safety profiles. In addition, capecitabine and panitumumab administration every three weeks demonstrated efficacy and safety in a geriatric population in the Panel study (32).

Despite these controversies, three-week mCAPIRI-P has several advantages including convenient oral administration, less frequent clinic attendance, and the absence of oxaliplatin-associated disturbing peripheral neuropathy. It is conceivable that randomized studies would unlikely be open to compare this chemotherapy regimen with other regimens. This report demonstrates that mCAPIRI-P is both safe and effective. The results are of particular importance, with the expected wide adoption of doublet chemotherapy with anti-EGFR antibody in the first-line setting, following the latest evidence from the PARADIGM (7) and TRIPLETE (8) trials.

In addition, with prolonged survival in colorectal cancer patients, the interest in studying CEA as a surrogate marker to reflect treatment response has been increasing (33–35). CEA kinetics analysis was consistent with the findings of a previous report (11) on the prognostic value of CEA response rate. Furthermore, multivariable analyses showed that patients with elevated baseline CEA levels achieved CEA normalization and/or were CEA responders had better PFS_OT_ and OS. The report on CEA kinetics in patients receiving mCAPIRI-P will further supplement the overall body of evidence.

The major limitation of this study is the retrospective study design. Non-laboratory toxicities can be potentially under-recorded. In addition, B-Raf was not routinely checked in the cohort. Finally, a long follow-up period is required to precisely estimate the survival data.

To conclude, mCAPIRI-P is an effective first-line treatment regimen for RAS wild-type advanced colorectal cancer in a real-world setting. It is generally safe with tolerable toxicity. Further studies are required to confirm these results.

## Data availability statement

The original contributions presented in the study are included in the article/**Supplementary Material**. Further inquiries can be directed to the corresponding author.

## Ethics statement

The studies involving human participants were reviewed and approved by New Territories West Cluster, Hospital Authority, Hong Kong. The ethics committee waived the requirement of written informed consent for participation.

## Author contributions

PY, SL, and FL contributed to conception and design of the study. PY organized the database. PY performed the statistical analysis. PY and SL wrote the first draft of the manuscript. All authors contributed to the article and approved the submitted version.
