# Supplementary material for: Effectiveness and safety of capecitabine, irinotecan and panitumumab in advanced colorectal cancer
Source: Front Oncol. 2023 Apr 6;13:1138357. doi: 10.3389/fonc.2023.1138357 (PMC10116611; doi:10.3389/fonc.2023.1138357)
Supplement: Supplementary file 1 [file Table_1.docx]

Supplementary Material

Effectiveness and Safety of Capecitabine, Irinotecan and Panitumumab in Advanced Colorectal Cancer

Pui Lam Yip, Wai Him Brian Fung, Ann Shing Lee, Chak Fei Lee, Sean Man Natalie Wong, Shing Fung Lee^*^

*** Correspondence:** Shing Fung Lee: [leesf@nuhs.edu.sg](mailto:leesf@nuhs.edu.sg)

Supplementary Data

**Supplementary Table 1.** Treatment results of landmark trials for RAS wild-type patients.

| First-line systemic treatment | | | | | |
| --- | --- | --- | --- | --- | --- |
|  | Our Cohort | PARADIGM (1) | PRIME (2) | IMPROVE (3) | CRYSTAL (4) |
| Treatment regimens | mCAPIRI-P | mFOLFOX-P | FOLFOX-P | FOLFIRI-P | FOLFIRI-C |
| PFS, months (95% CI) | - | 12.9 (11.3-13.6) | 10.0 (9.3-11.4) | - | 9.9 (9.0 to 11.3) |
| PFS_OT,_ months (95% CI) | 15.4 (12.5-18.3) | - | - | 17.6 (7.5-27.8) | - |
| 1-year PFS_OT_, % (95%CI) | 61.9(52.5%-71.3) | - | - | 61.3 | - |
| OS, months (95% CI) | 25.5 (17.6-33.4)  (data immature) | 36.2 (32.0-39.0) | 23.9 (20.3-27.7) | NR | 23.5 (21.2-26.3) |
| Conversion chemotherapy | | | | | |
|  | Our Cohort | CELIM (5) | Ye et al. (6) | TRIPLETE (7) | VOLFI (8) |
| Treatment regimens | mCAPIRI-P | FOLFIRI/FOLFOX-C (All RAS^a^) | FOLFIRI/FOLFOX-C | FOLFOX-P (liver-only subgroup) | FOLFOXIRI-P |
| Secondary resection/ Liver treatment, % | 51.6 | 50 | - | - | 33 |
| R0 resection | 32.3 | 34 | 25.7 | 43 | - |

^a^Data presented was RAS unselected patient population

Abbreviations: CAPIRI, capecitabine-irinotecan; C, cetuximab; CI, confidence interval; FOLFOX, 5-fluorouracil-oxaliplatin; FOLFIRI, 5-fluorouracil-irinotecan; FOLFOXIRI, 5-fluorouracil-oxaliplatin-irinotecan; m, modified; NR, not reported; P, panitumumab; PFS_,_ progression-free survival; PFS_OT,_ progression-free survival on treatment

**References:**

1. Yoshino T, Watanabe J, Shitara K, Yasui H, Ohori H, Shiozawa M, et al. Panitumumab (PAN) plus mFOLFOX6 versus bevacizumab (BEV) plus mFOLFOX6 as first-line treatment in patients with RAS wild-type (WT) metastatic colorectal cancer (mCRC): Results from the phase 3 PARADIGM trial. Journal of Clinical Oncology. 2022;40(17_suppl):LBA1-LBA.

2. Douillard JY, Siena S, Cassidy J, Tabernero J, Burkes R, Barugel M, et al. Final results from PRIME: randomized phase III study of panitumumab with FOLFOX4 for first-line treatment of metastatic colorectal cancer. Annals of oncology : official journal of the European Society for Medical Oncology. 2014;25(7):1346-55.

3. Avallone A, Giuliani F, Nasti G, Montesarchio V, Santabarbara G, Leo S, et al. Randomized intermittent or continuous panitumumab plus FOLFIRI (FOLFIRI/PANI) for first-line treatment of patients (pts) with RAS/BRAF wild-type (wt) metastatic colorectal cancer (mCRC): The IMPROVE study. Journal of Clinical Oncology. 2022;40(16_suppl):3503-.

4. Van Cutsem E, Köhne CH, Láng I, Folprecht G, Nowacki MP, Cascinu S, et al. Cetuximab plus irinotecan, fluorouracil, and leucovorin as first-line treatment for metastatic colorectal cancer: updated analysis of overall survival according to tumor KRAS and BRAF mutation status. Journal of clinical oncology : official journal of the American Society of Clinical Oncology. 2011;29(15):2011-9.

5. Folprecht G, Gruenberger T, Bechstein WO, Raab HR, Lordick F, Hartmann JT, et al. Tumour response and secondary resectability of colorectal liver metastases following neoadjuvant chemotherapy with cetuximab: the CELIM randomised phase 2 trial. The Lancet Oncology. 2010;11(1):38-47.

6. Ye LC, Liu TS, Ren L, Wei Y, Zhu DX, Zai SY, et al. Randomized controlled trial of cetuximab plus chemotherapy for patients with KRAS wild-type unresectable colorectal liver-limited metastases. Journal of clinical oncology : official journal of the American Society of Clinical Oncology. 2013;31(16):1931-8.

7. Rossini D, Antoniotti C, Lonardi S, Pietrantonio F, Moretto R, Antonuzzo L, et al. Upfront Modified Fluorouracil, Leucovorin, Oxaliplatin, and Irinotecan Plus Panitumumab Versus Fluorouracil, Leucovorin, and Oxaliplatin Plus Panitumumab for Patients With RAS/BRAF Wild-Type Metastatic Colorectal Cancer: The Phase III TRIPLETE Study by GONO. Journal of clinical oncology : official journal of the American Society of Clinical Oncology. 2022;40(25):2878-88.

8. Modest DP, Martens UM, Riera-Knorrenschild J, Greeve J, Florschütz A, Wessendorf S, et al. FOLFOXIRI Plus Panitumumab As First-Line Treatment of RAS Wild-Type Metastatic Colorectal Cancer: The Randomized, Open-Label, Phase II VOLFI Study (AIO KRK0109). Journal of clinical oncology : official journal of the American Society of Clinical Oncology. 2019;37(35):3401-11.
